# Kininogen Level in the Cerebrospinal Fluid May Be a Potential Biomarker for Predicting Epileptogenesis

**DOI:** 10.3389/fneur.2019.00037

**Published:** 2019-01-31

**Authors:** Jing Zou, Xinxin Wang, Ligang Huang, Juan Liu, Yingying Kong, Shengtian Li, Qinchi Lu

**Affiliations:** ^1^Department of Neurology, Renji Hospital, School of Medicine, Shanghai Jiao Tong University, Shanghai, China; ^2^Key Laboratory for the Genetics of Development and Neuropsychiatric Disorders (Ministry of Education), Shanghai Key Laboratory of Psychotic Disorders, and Brain Science and Technology Research Center, Bio-X Institutes, Shanghai Jiao Tong University, Shanghai, China

**Keywords:** biomarker, encephalitis, epilepsy, epileptogenesis, kininogen, pilocarpine, proteomics

## Abstract

**Purpose:** Epilepsy is a highly disabling neurological disorder. Brain insult is the most critical cause of epilepsy in adults. This study aimed to find reliable and efficient biomarkers for predicting secondary epilepsy.

**Materials and methods:** The LiCl-pilocarpine (LiCl-Pilo) chronic epilepsy rat model was used, and rat cerebrospinal fluid (CSF) was collected 5 days after status epilepticus (SE). The CSF was analyzed using the label-free LC-ESI-Q-TOF-MS/MS. Differential expression of proteins was confirmed using enzyme-linked immunosorbent assay (ELISA) and Western blotting. The corresponding protein level in the CSF of patients with encephalitis in the postacute phase was determined using ELISA and compared between patients with and without symptomatic epilepsy after encephalitis during a 2-year follow-up.

**Results:** The proteomics and ELISA results showed that the protein level of kininogen (KNG) was obviously elevated in both CSF and hippocampus, but not in serum, 5 days after the onset of SE in LiCl-Pilo chronic epilepsy model rats. In patients with encephalitis, the protein level of KNG in the CSF in the postacute phase was significantly elevated in patients with a recurrent epileptic seizure during a 2-year follow-up than in patients without a recurrent seizure.

**Conclusion:** KNG in the CSF may serve as a potential biomarker for predicting epileptogenesis in patients with encephalitis.

## Introduction

Epilepsy is a highly disabling neurological disorder characterized by recurrent epileptic attacks (1). Repeated epileptic attacks are often accompanied by movement, sensation, endocrine, cognitive, and psychological disorders (2), further posing an economic and emotional burden to both patients and communities. Epilepsy control is still far from perfection despite the emergence of new drugs and therapies, and nearly a quarter of patients suffer from drug-resistant epilepsy (3). In adults, epilepsy usually results from various brain insults, for example, trauma, encephalitis, and stroke. In case of acquired epilepsy, the term “epileptogenesis” describes an injury-initiated change that causes surviving neuron populations to generate abnormal, synchronous, and recurring epileptiform discharges that produce focal or generalized behavioral seizures (4). Epileptogenesis period refers to a time period between the insult and the occurrence of the first unprovoked seizure (5). As clinicians are unable to figure out which patient will develop spontaneous epilepsy secondary to the aforementioned brain insults, antiepileptic treatment usually starts after recurrent seizure attacks, which may occur weeks, months, or years after primary brain insult. Therefore, if effective nontraumatic biomarkers for epilepsy can be evaluated prior to significant seizure attacks, it can reduce the risk of recurrent seizure attacks and improve the prognosis. Thus, in recent years, the exploration for biomarkers of epileptogenesis has become one of the major focuses in epilepsy research field (6, 7).

The expression levels of certain miRNAs (8) and a series of mRNAs were found to be upregulated with the use of peripheral blood of epileptic animals as a sample (9, 10). However, to what extent the changes in peripheral blood reflect the changes in brain network function remains unknown.

Compared with peripheral blood, the cerebrospinal fluid (CSF) better reflects pathological changes in the brain. Lumber puncture has long been proved to be a safe and essential examination in many neurological disorders, especially in inflammation and infectious brain diseases, which are also common causes for symptomatic epilepsy. In patients with traumatic brain injury, the levels of C-tau in the CSF were used to predict the long-term mortality, motility, and cognitive function after traumatic brain injury (11–14). Yet, these studies did not use epilepsy as an outcome event. Proteomics analysis showed increased levels of vitamin D-binding protein tetranectin and decreased levels of clusterin in CSF in patients with temporal lobe epilepsy (TLE), suggesting that they might serve as a potential biomarker of drug-resistant epilepsy (15, 16). However, whether proteins found to be altered in patients with established epilepsy began to change during epileptogenesis is unknown; in other words, they might not serve as predictors of epilepsy. An ideal timing to explore the biomarkers of epilepsy is when brain insults happen, yet recurrent epilepsy attacks have not occurred, that is, the epileptogenesis period (17).

In the present study, the proteomic technique was used to analyze changes in protein expression levels in the CSF 5 days after the onset of status epilepticus (SE) in the LiCl-pilocarpine (LiCl-Pilo) chronic rat epilepsy model. After evaluating the occurrence of spontaneous recurrent seizures (SRS) by video monitoring during 6 weeks after the onset of SE, the differently expressed proteins in the CSF between naïve control and SRS-detected LiCl-Pilo rats were screened. Validation by enzyme-linked immunosorbent assay (ELISA) and Western blotting indicated that the expression level of kininogen (KNG) was significantly upregulated in the CSF of rats with SRS. Further, the CSF was collected from a group of patients with encephalitis in the postacute phase, and a 2-year follow-up was conducted to verify whether the patients developed SRS. This study demonstrated that patients with secondary epilepsy showed a significantly higher KNG level in the postacute phase compared with the levels in patients with encephalitis who did not develop SRS and in control patients. Taken together, the results suggested that the KNG level in the CSF might serve as a potential biomarker of early epileptogenesis.

## Materials and Methods

### Animals

A total of 84 male adult Sprague–Dawley (SD) rats were bought from Shanghai Laboratory Animal Center. They were housed in a controlled laboratory environment with regular animal chow and water *ad libitum* and maintained under a reversed 12-h light/dark cycle (light on at noon). Particular efforts were made to minimize the number of animals used and the potential for animal suffering.

### Seizure Induction

The rats were housed for 1 week prior to the study to minimize the potential stress of human interaction. All groups of rats were of similar weight (200 ± 10 g) and age. The rats in the LiCl-Pilo group were injected intraperitoneally with LiCl (127 mg/kg, Sigma, USA) 18 h before seizure induction. Atropine sulfate (1 mg/kg, Shanghai General Pharmaceutical Co., Shanghai, China) was injected intraperitoneally 30 min before seizure induction. Finally, a single dose of pilocarpine (40 mg/kg, Sigma, USA) was administered to induce seizures. Rat behavior was scored according to the Racine scale (18). SE was defined as continuous stage 4 or more serious seizures, which lasted for more than 30 min. SE was terminated by injecting chloral hydrate (300 mg/kg, Sigma, USA). The operation in the LiCl group was similar to that in the LiCl-Pilo group, except that pilocarpine was replaced by normal saline. The naïve group did not receive any operation.

### CSF Extraction

Five days after SE, the CSF of rats was collected as described in a previous study (18). The rats were anesthetized by injecting pentobarbital sodium (1 mg/kg, Sigma, USA) intraperitoneally and mounted in a stereotaxic frame (51600, Stoelting Co, USA). The skin over the cisterna magna was shaved and disinfected with povidone–iodine. A midline sagittal incision was made over the dorsal aspect of the hindbrain, and three layers of muscle were carefully peeled back to expose the cisterna magna. About 60 μL of colorless and clear CSF was extracted slowly using a syringe needle. The skin wounds were sewed up and disinfected properly. The rats were injected subcutaneously with 1 mL of normal saline to prevent dehydration and kept warm before awake from anesthesia. The CSF was stored at –80°C for further experiments.

### Video Monitoring of Spontaneous Recurrent Seizures (SRS)

Six weeks after the model establishment, the rats in the LiCl-Pilo group that survived from CSF collection were subjected to dynamic video surveillance for 3–6 days. The rats with SRS were screened. The seizure scale was classified according to the Racine scale. The duration and frequency of seizures were recorded.

### Preparation of Tryptic Digests

Each sample of 15 μl CSF was diluted using 55 μl 100 mM NH4HCO3 and 10 μl 100 mM DTT at 60°C for 1 h. After incubation, 10 μl of 450 mM (83.23 mg/ul) was added for carboxymethylation, and the sample was allowed to incubated for 30 min in dark. Protein digestions were conducted over with trypsin (100 ng/μl) in a 1:20 trypsin-to-protein mass-ratio. Digestion was performed overnight at 37°C and further incubated at 56°C for another 20 min in the next day morning. The tryptic peptides were dried using Savant SpeedVac (Thermo Scientific) and resolved in 20 μl 0.1% formic acid. Sample was then desalted and purified individually by 10 μl ZipTip pipette tip system (Millipore). Protein attached to the resin in Ziptip was dissolved in 20 μl eluting buffer (80% acetonitrile + 0.1% formic acid+ H_2_O) and dried by Savant Speedvac. Finally, sample was reconstituted to 40 μl (2% acetonitrile + 0.1% formic acid + H_2_O) and 20 μl was analyzed by Nano LC-MS/MS.

### Nano LC-MS/MS

Each sample was analyzed using an LC system (Nano Pump, Ultimate 3000, Dionex, Thermofisher) coupled with an ESI-Q-TOF mass spectrometer (maxis,Impact, Bruker Daltonik, Germany). Each peptide sample was separated using a solvent system with solvent A consisting of 99.9% water and 0.1% formic acid, and solvent B consisting of 99.9% acetonitrile and 0.1% formic acid. The peptides were eluted with gradients 2–20% B in 75 min, 20–80% B in 15 min, 80% hold for 15 min, 2% hold for 15 min with a constant flow rate of 350 nl/min. The LC setup was coupled online to a Q-TOF using a nano-ESI source (Bruker Daltonik, Germany) in data dependent acquisition mode (m/z 350~1,500). The Source Capillary was set at 2400 v. the flow and temperature of dry gas was 2.0 1/min and 120°C respectively. The mass spectrometer was set as one full MS scan followed by 10 MS/MS scans on the 10 most intense ions from the MS spectrum.

### MS Data Analysis

Tandem mass spectra were processed using PEAKS Studio version 7.5 (Bioinfor Inc. CA). PEAKS DB was set up to search the uniprot_2016_02 Rat database (29982 entries) assuming the digestion enzyme Trypsin. PEAKS DB was searched with a fragment ion mass tolerance of 0.050 Da and a parent ion tolerance of 15.0 PPM. Carbamidomethyl of cysteine was specified as a fixed modification. Deamidated of asparagine and glutamine and oxidation of methionine were specified in Mascot as variable modifications. Peptides were filter by FDR 1%. PEAKS Q was used for peptide and protein abundance calculation. Normalization was performed on total ion chromatogram. Medians were used for averaging. Different expressed proteins were filtered if their fold change were over 1.5 fold and PEAKS $$significance−10logP-value over 15.

### Hippocampus and Serum Collection

The bilateral hippocampus and serum were collected from another parallel group of rats to compare the protein levels in hippocampus, and serum. Five days after model establishment, the rats were quickly decapitated under deep isoflurane anesthesia. The peripheral blood was collected from neck vessels and centrifuged (3,000 g, 10 min, 4°C). The serum was finally aliquoted and frozen (–) for subsequent analysis. The intact brain was promptly removed and placed in ice. The bilateral hippocampus was separated and stored at −80°C for further experiments.

### Enzyme-Linked Immunosorbent Assay (Elisa)

The KNG protein level in the CSF and serum was tested using commercial ELISA kits, Rat Kininogen ELISA Kit (ab157742) and Human Kininogen ELISA Kit (ab108876) (Abcam, UK). Sample analysis was conducted according to the instruction provided. Samples were diluted using a 1 × diluent solution. Dilution ratio in the rat sample was 1:20 (CSF) and 1:10000 (plasma). Dilution ratio in the human sample was 1:100 (CSF) and 1:20000 (plasma).

### Western Blotting Analysis

The hippocampal tissues were homogenized on ice in RIPA buffer (Sigma–Aldrich, USA) using the auto TissueLyser-24 (Shanghai Jingxin, China) (2 × 30 s). Complete Protease Inhibitor Cocktail (Roche, Germany) was added into the RIPA buffer. Tissue homogenates were centrifuged (12,000 g, 30 min, 4°C), and the supernatants were finally aliquoted and frozen (–) for subsequent analysis.

Using the bicinchoninic acid protein assay kit (Sangon Biotech, China), the concentration of all protein extracts was measured. Samples, each containing 30 μg of total protein, were loaded and separated by electrophoresis with 6% sodium dodecyl sulfate–polyacrylamide gel electrophoresis. The proteins were then electrotransferred to polyvinylidene difluoride membranes (Millipore, USA). The membranes were then blocked in 5% bovine serum albumin (BSA) or 5% non-fat milk. BSA or non-fat milk was dissolved in 1 × TBST (140 mM NaCl, 3 mM KCl, 25 mM Tris, and 0.1% Tween 20). After blocking, the membranes were incubated with primary antibody overnight at 4°C. Antibody dilutions were 1:1000 for KNG (rabbit polyclonal antibody; Abcam, UK) and 1:5000 for β-actin (rabbit monoclonal antibody; Cell Signaling Technology, USA). After incubation, the membranes were washed in 1 × TBST (3 × 10 min) with primary antibodies and then incubated with horseradish peroxidase–conjugated goat antirabbit immunoglobulin G (1:5000, HSA003) for 2 h at room temperature. The secondary antibody was diluted in 5% non-fat milk. The membranes were then rewashed with 1 × TBST (3 × 10 min). Finally, the protein bands were detected using an enhanced chemiluminescent reagent, and ImageJ software (NIH, USA) was used to quantify the intensity of each band. Relative protein levels were determined using the ratio of the band intensity of the target protein to that of its respective β-actin loading control.

### Patients Recruitment and CSF Collection

Twelve patients with encephalitis in Renji Hospital affiliated to Shanghai Jiao Tong University School of Medicine were enrolled. Five patients with functional disorders served as controls. Written consents for participation in the study were obtained from patients. The following inclusion criteria were applied according to the previous studies (19, 20): (1) consisted with the clinical manifestation of encephalopathy: consciousness changes lasted more than 24 h, the appearance of indifference, irritability or personality and behavior changes. (2) two or more of the following manifestations: (a) fever (≥38°C); (b) seizures; (c) focal neurological signs; (d) CSF leukocytes ≥ 5/per high magnification; (e) electroencephalography(EEG) abnormalities were consistent with encephalitis changes; (f) neuroimaging suggested changes in encephalitis. (3) no epilepsy history.

As CSF tests are essential in encephalitis diagnosis, the collection of CSF is easy and harmless to patients. The CSF of last lumbar puncture before discharge of patients was collected. At that time, encephalitis was well-controlled after proper therapy. Routine tests of CSF, including cell number, protein level, and glucose, recovered to normal, and the patient was ready for discharge. The acute phase of encephalitis was over, yet spontaneous recurrent symptomatic epilepsy did not appear in most patients. This timing, “the postacute phase,” was approximate to what was defined as epileptogenesis period in a chronic epilepsy animal model (21). The CSF was centrifuged (3,000 g, 30 min, 4°C), and the supernatants were finally aliquoted and frozen (−80°C) for subsequent analysis.

Patients were followed up for 2 years. Symptomatic epilepsy secondary to encephalitis was diagnosed if unprovoked epileptic seizure appeared during follow-up, regardless of seizure occurrence during acute phase.

### Statistical Analysis

Data were expressed as means ± standard error of the mean (SEM). Statistical analysis was conducted using the *t*-test or one-way analysis of variance (ANOVA) with a significance level of *P* < 0.05.

## Results

### Screening of Proteins in the CSF of SD Rats That Changed During Early Epileptogenesis

Among the 53 rats in the LiCl-Pilo group, 28 met the standards defined by SE. Five rats died and 23 survived 5 days after SE. After CSF collection, nine (9/23) rats died. One rat was excluded because of blood contamination of CSF. The numbers of rats in all three groups enrolled in CSF collection are shown in Table 1.

**Table 1:** Numbers of rats enrolled in CSF collection (*n*).

**Groups**	**Pre-collection**	**Death (mortality)**	**CSF blood contamination**	**Survived rats**
LiCl-Pilo	23	9 (39.13%)	1	13
LiCl	20	6 (30%)	2	12
Naïve	11	2 (18.18%)	1	8

The 13 rats in LiCl-Pilo group were video monitored 6 weeks after SE for 3–6 days. A total of 66 attacks of Racine 2 or more serious seizures were observed. The average seizure frequency was 1.25 ± 1.14 per day. The numbers of epileptic attacks (Racine 2 or severer) during video monitoring in each rat are displayed in Table 2. One rat (#4), which failed to show SRS in video monitoring, was excluded to ensure that all rats enrolled in proteomics analysis displayed SRS.

**Table 2:** Number of epileptic attacks (Racine 2 or severer) during video monitoring.

**Rat number**	**Day 1**	**Day 2**	**Day 3**	**Day 4**	**Day 5**	**Day 6**	**Mean**
1	3	2	1	8	3	0	2.83
2	3	0	0	0	3	1	1.17
3	0	0	0	1	/	/	0.25
4	0	0	0	0	/	/	0
5	2	3	2	0	/	/	1.75
6	1	0	0	0	/	/	0.25
7	6	2	3		/	/	3.67
8	0	0	2	0	/	/	0.5
9	1	1	0	1	/	/	0.75
10	0	7	1	/	/	/	2.67
11	0	3	0	/	/	/	1
12	1	1	0	1	/	/	0.75
13	0	0	2	1	/	/	0.75

**Figure 1:**
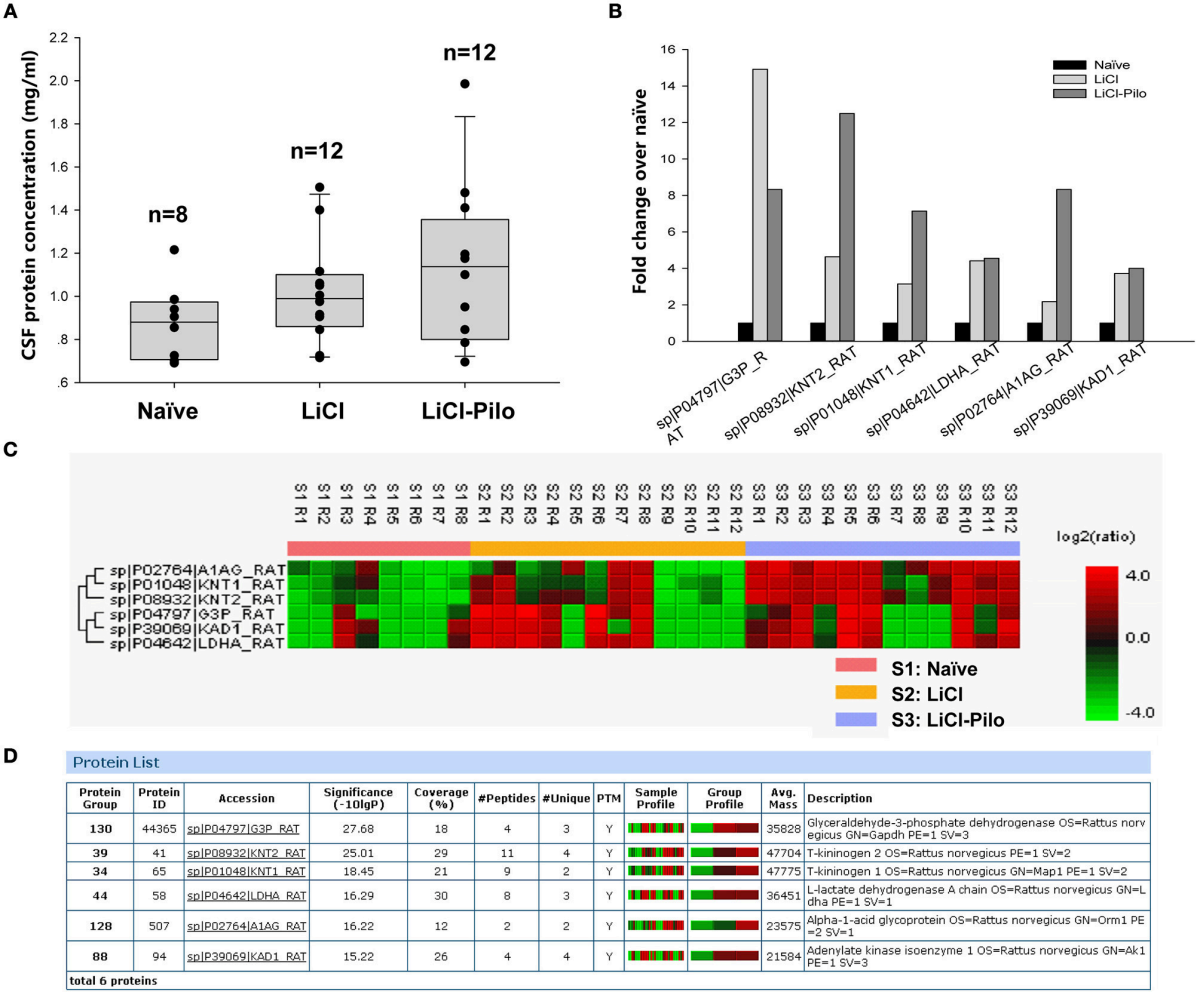
Proteomic analysis of CSF in SD rats 5 days after SE. **(A)** The concentration of total proteins in the Naïve, LiCl and LiCl-Pilo groups. **(B)** The fold changes in proteins differentially expressed in the CSF of the three groups. The protein in the naïve group was normalized to 1.0. **(C,D)** Results of proteomics analysis.

After confirming SRS of 12 rats in the LiCl-Pilo group, this study aimed to compare the changes in protein expression in the CSF among LiCl-Pilo, LiCl, and naïve control rats. As shown in Figure 1A, the concentration of total proteins in the three groups was 1.13 ± 0.11 mg/mL (LiCl-Pilo, *n* = 12), 1.02 ± 0.07 mg/mL (LiCl, *n* = 12), and 0.88 ± 0.06 mg/mL (naïve, *n* = 8) (*p* = 0.161, one-way ANOVA). Subsequent proteomic analysis detected 146 proteins; 6 of them were differently expressed (Figure 1B listed the fold changes in proteins differentially expressed in the CSF of the three groups. The protein in the naïve group was normalized to 1.0.). Among them, the expression levels of T-kininogen (KNG) 1 and 2 and alpha-1-acid glycoprotein significantly increased in the LiCl-Pilo group compared with both naïve and LiCl groups (Figures 1C,D). The original data of proteomic analysis is provided in Supplementary Material.

### Verification of the Upregulation of KNG in the Rat CSF During Early Epileptogenesis

As a vital component of kallikrein–kinin system (KKS) (22), KNG is hydrolyzed to bradykinin. As the involvement of bradykinin (23) and its receptors B_1_ and B_2_ in epileptogenesis has already been reported (24–26), this study examined the expression of KNG in CSF. Three kinds of KNG exist in rats: high-molecular-weight kininogen (H-KNG), low-molecular-weight kininogen (L-KNG), and T-KNG. Of these, T-KNG is similar to L-KNG in protein structure and function. In human, only L-KNG and H-KNG are present. Therefore, the overall KNG level rather than that of T-KNG alone was tested in both rats and patients.

**Figure 2:**
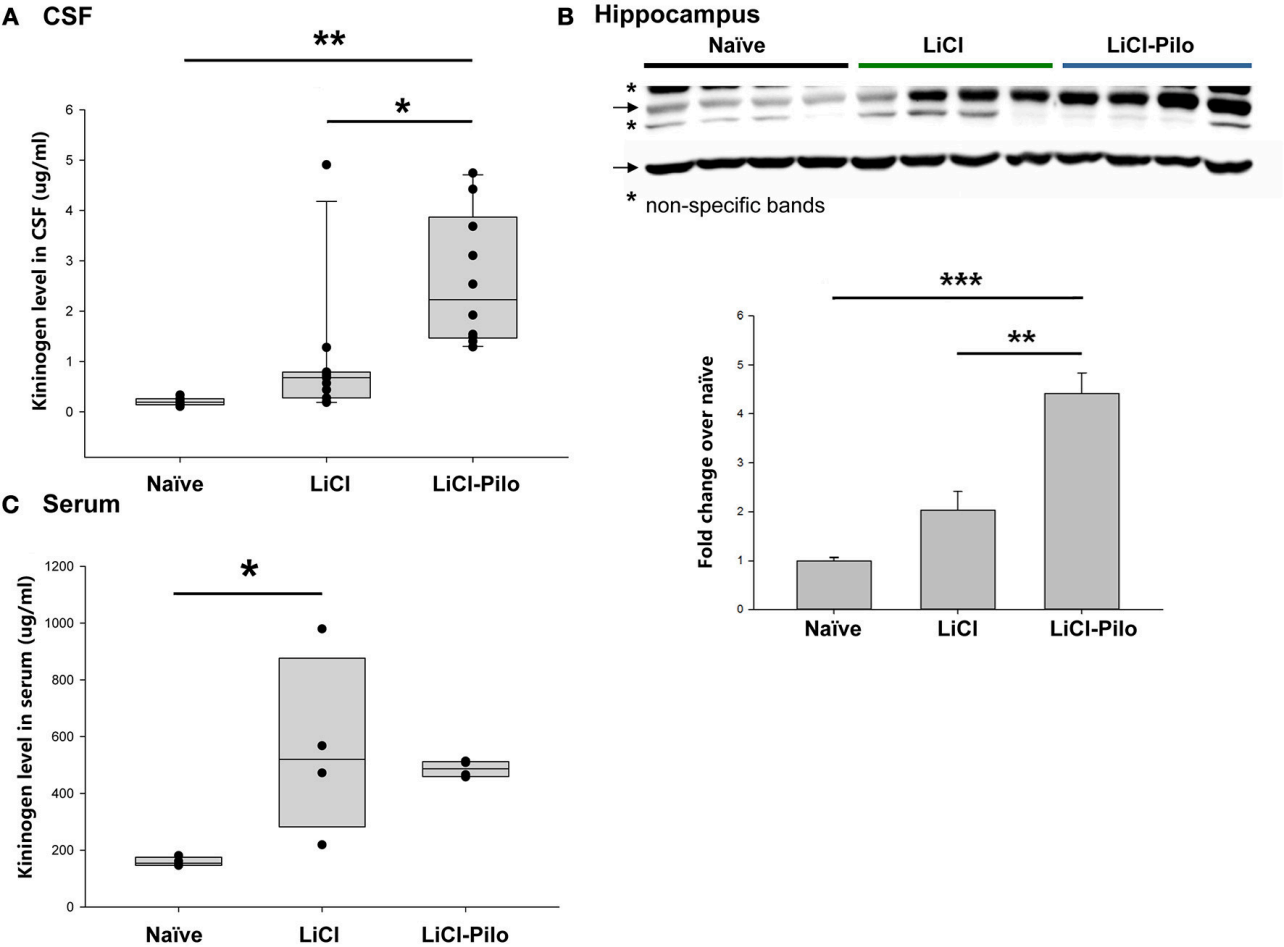
Verification of the upregulation of KNG in CSF during early epileptogenesis. **(A)** The level of KNG in CSF using ELISA. **(B)** The level of KNG in the hippocampus using Western blotting. **(C)** The level of KNG in serum using ELISA. ^*^p < 0.05, ^**^p < 0.01, ^***^p < 0.001.

Using the ELISA technique, the level of KNG in the identical samples used for proteomic analysis was examined. Since 4 samples were used up after proteomic analysis, a total of 28 samples were applied in ELISA test. The level of KNG was found to be significantly higher in the LiCl-Pilo group (*n* = 10, 2.61 ± 0.41 μg/ml) than in the LiCl (*n* = 11, 0.98 ± 0.40 μg/ml) (^*^p = 0.031, one-way ANOVA) and naïve groups (*n* = 7, 0.21 ± 0.3 μg/ml) (^**^p = 0.001, one-way ANOVA) (Figure 2A). Similar to the result of proteomics, the ratio of KNG in the three groups was 1:4.67:12.43 (naïve:LiCl:LiCl-Pilo).

The level of KNG in the hippocampus was examined using Western blotting and in peripheral blood using ELISA technique to explore the source of KNG in CSF in another group of rats. In the hippocampus, the level of KNG was significantly higher in the LiCl-Pilo group (*n* = 4, 4.42 ± 0.84 fold) than in the LiCl (*n* = 4, 2.03 ± 0.76 fold) (*p* = 0.002, one-way ANOVA) and naïve groups (*n* = 4) (*p* < 0.001, one-way ANOVA) (Figure 2B, the original Western blotting image can be accessed in Supplementary Material). In contrast, the serum expression levels of KNG in the LiCl-Pilo group was not significantly different from that in the LiCl group, although the level of KNG in the LiCl group was significantly higher than that in the naïve group (Figure 2C; *n* = 4 for each group, LiCl-Pilo 486.12 ± 14.35 μg/ml; LiCl, 559.38 ± 158.11 μg/ml; naïve 158.72 ± 7.92 μg/ml). The increased CSF level of KNG was concomitant with enhanced expression in the hippocampus but not in peripheral blood, indicating that this upregulation was due to an increased synthesis in brain tissue rather than peripheral synthesis.

### Correlation of Postacute Phase KNG Level in the CSF and Symptomatic Epilepsy in Patients With Encephalitis

A total of 12 patients with encephalitis were further enrolled from December 2013 to April 2014 to verify whether the increase in the KNG level in the CSF was related to epileptogenesis in humans. The clinical manifestations and laboratory examinations of encephalitis patients are shown in Table 3. Five patients (4 females, 1 male) of functional disorder were enrolled as control, including 3 tension-type headache, 1 depression disorder and 1 schizophrenia patients, aged 19-39 years old.

**Table 3:** Clinical manifestations and laboratory test results of patients with encephalitis.

**Patient number**	**Gender**	**Age**	**Encephalopathy**	**Fever**	**Seizure**	**Neurology signs**	**Elevated CSF leukocytes**	**EEG**	**Neuro-imaging**	**Diagnosis**	**SRS in follow-up**
1	F	27	+	+	GTCS, Myocl, SPS, PS	+	+	Diffuse abnormity	–	Anti-NMDAR encephalitis	SPS, CPS
2	M	36	+	+	–	–	+	–	Bilateral frontal and temporal lobe lesions	Bacterial meningoe-ncephalitis	SPS
3	M	49	+	+	–	–	+	–	–	Viral encephalitis	–
4	M	17	+	+	GTCS	–	+	–	–	Viral encephalitis	–
5	M	36	+	+	GTCS	–	+	Diffuse abnormity	Bilateral temporal and occipital lobe lesion with enhancement	Viral encephalitis	–
6	M	22	+	+	–	+	–	Diffuse abnormity	–	Viral encephalitis	–
7	F	24	+	+	GTCS, SPS, CPS	+	+	Diffuse abnormity	Bilateral frontal, temporal lobe lesion	Viral encephalitis	GTCS,SPS,CPS
8	M	77	+	+	SPS, Myocl	–	+	Diffuse abnormity	–	Viral encephalitis	SPS
9	M	28	+	+	GTCS, Myocl, SPS, CPS	–	+	Diffuse abnormity	–	Viral encephalitis	GTCS,Myocl,SPS,CPS,AB
10	F	46	+	+	–	–	+	Diffuse abnormity	–	Viral encephalitis	–
11	M	36	+	+	GTCS	–	+	–	–	Viral encephalitis	–
12	M	32	+	+	–	–	+	–	–	Viral encephalitis	–

*SPS, single partial seizure; CPS, complex partial seizure; GTCS, generalized tonic-clonic seizure; Myocl, Myoclonic; AB, absence attack*.

#### The Level of KNG in CSF and Serum of Encephalitis Patients

**Figure 3:**
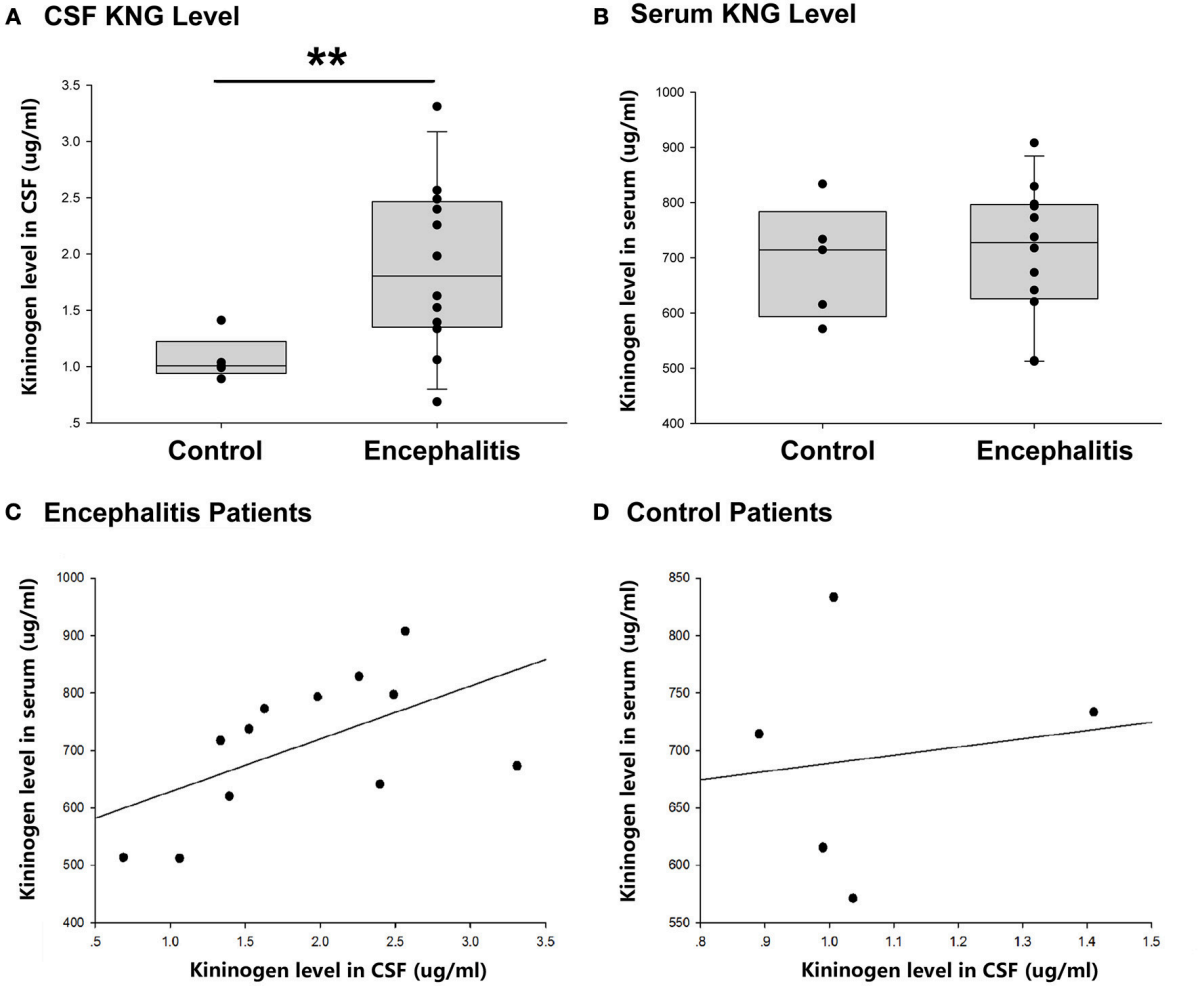
CSF and serum KNG protein level of encephalitis patients and controls. **(A)** The level of KNG in CSF of encephalitis and control patients. **(B)** The level of KNG in serum of encephalitis and control patients. **(C)** The correlation of serum and CSF KNG levels in encephalitis patients. **(D)** The correlation of serum and CSF KNG levels in control patients. ^**^p < 0.01.

As shown in Figure 3A, the level of KNG in CSF of encephalitis patients (1.89 ± 0.22 μg/ml, *n* = 12) was significantly higher than that in controls (1.07 ± 0.09 μg/ml, *n* = 5) (^**^p < 0.01, *t*-test). However, the level of KNG in serum of encephalitis patients (693.55 ± 46.21 μg/ml, *n* = 12) was not significantly different than that in controls (709.68 ± 35.30 μg/ml, *n* = 5) (*p* > 0.05, *t*-test) (Figure 3B).

The correlation of serum and CSF KNG levels was higher in patients with encephalitis (Pearson coefficient 0.562, *p* = 0.057) than in controls (Pearson coefficient 0.138, *p* = 0.825), but both failed to showed significance (Figures 3C,D), suggesting that the upregulated KNG level in the CSF was not likely due to increased synthesis in peripheral tissues, in accordance with the finding in the study on rats.

#### The Correlation Between Early Epileptogenesis Period CSF and Serum KNG Level and Acute Phase Symptomatic Seizures

**Figure 4:**
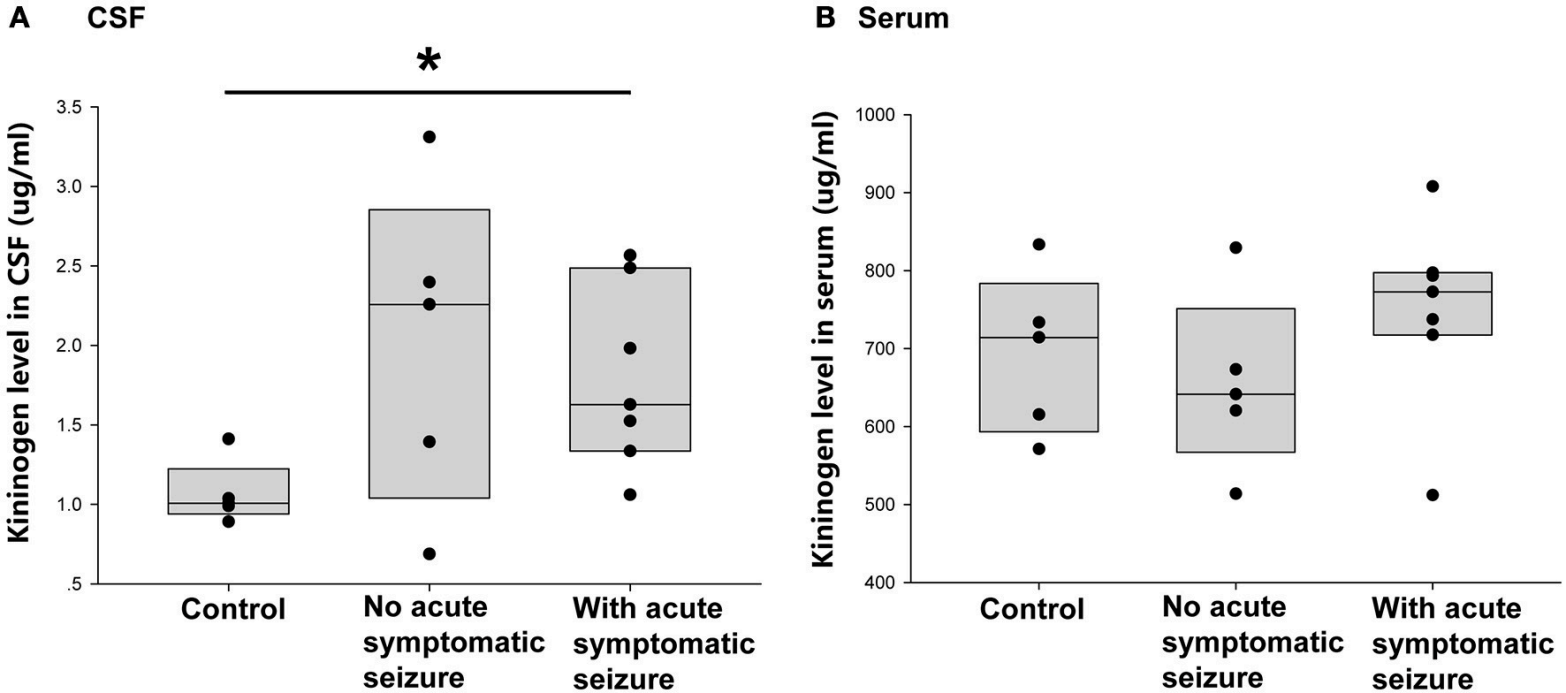
Correlation between acute symptomatic seizure and KNG protein level in post-acute phase CSF/ serum. **(A)** The level of KNG in CSF of control and encephalitis patients with and without acute phase symptomatic seizure. **(B)** The level of KNG in serum of control and encephalitis patients with and without acute phase symptomatic seizure. ^*^p < 0.05.

Of the 12 patients with encephalitis, seven had symptomatic seizures during acute phase. Thus, the next question was whether the occurrence of acute phase symptomatic seizure had an impact on the increased CSF KNG level in the postacute phase. As shown in Figure 4A, the KNG level in CSF of two groups of encephalitis patients were not significantly different with each other (*p* = 0.962, one-way ANOVA), though higher than in controls(^*^p = 0.029, one-way ANOVA), indicating that the increased CSF KNG level in early epileptogensis period did not depend on the occurrence of seizures during acute phase. On the contrary, the serum KNG level in all three groups was not significantly different (Figure 4B. *p* > 0.05, one-way ANOVA).

#### The Correlation Between Early Epileptogenesis Period CSF's KNG Level and Symptomatic Epilepsy in 2 Years Follow-Up

**Figure 5:**
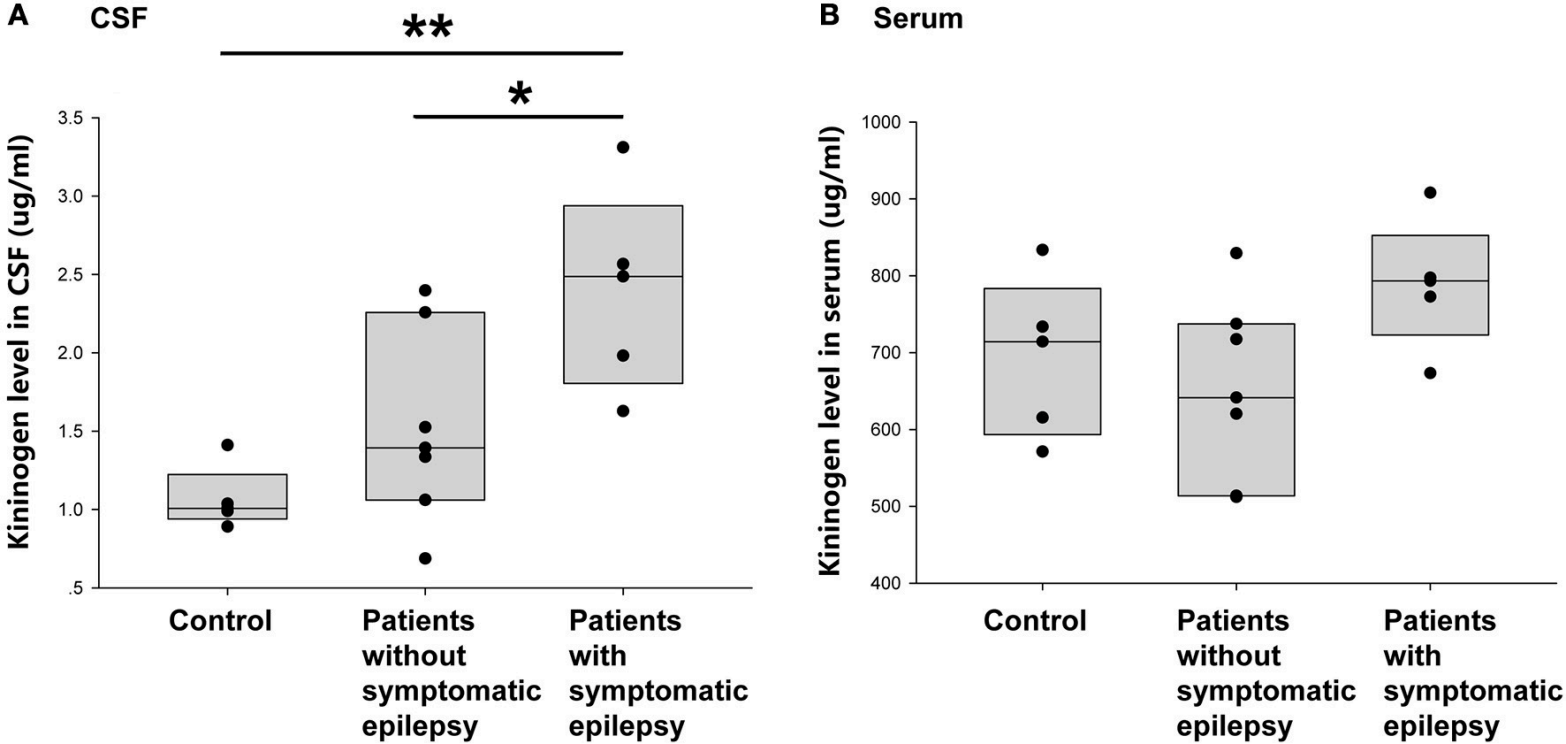
Correlation between post-acute phase CSF and serum KNG protein level and symptomatic epilepsy in 2 years. **(A)** The level of KNG in CSF of control and encephalitis patients with and without symptomatic epilepsy. **(B)** The level of KNG in serum of control and encephalitis patients with and without symptomatic epilepsy. ^*^p < 0.05, ^**^p < 0.01.

This study next compared the CSF KNG levels in controls and encephalitis patients with and without unprovked epilepsy in the 2-year follow-up to verify whether the development of secondary epilepsy after encephalitis was related to the KNG level in the CSF during the postacute phase. As shown in Figure 5A, the KNG level in the CSF was significantly higher in patients with secondary epilepsy (*n* = 5, 2.39 ± 0.29 μg/ml) than in patients without secondary epilepsy (*n* = 7, 1.52 ± 0.23 μg/ml) (*p* = 0.046, one-way ANOVA) or controls (*n* = 5, 1.07 ± 0.09 μg/ml) (*p* = 0.005, one-way ANOVA). On the contrary, the KNG level in serum was not significantly different among the three groups (Figure 5B). Taken together, the increased KNG level in the CSF in the postacute phase was related to unprovked epilepsy during the 2-year follow-up, but not related to acute phase seizures.

## Discussion

Increasing clinical evidences indicated that CSF proteins could be potential markers of CNS diseases, such as CSF 14-3-3 protein for Crearzfeldt-Jakob disease (27) and Aβ for Alzheimer's disease (28). Our results showed that CSF KNG level was elevated during early epileptogensis period both in LiCl-Pilo epilepsy model rats and in encephalitis patients who developed epilepsy in the 2-year follow-up period. These findings provided evidence that the elevation of KNG in the CSF might predict epileptogenesis. This was the first study based on both experimental and clinical observations showing that a protein in the CSF might serve as a potential biomarker of epileptogenesis.

### Use of CSF to Explore Biomarkers of Epileptogenesis

In this study, about 60 μL of CSF was collected from each model rat and analyzed using a label-free LC-ESI-Q-TOF-MS/MS. CSF studies in epilepsy animal models are rare because CSF collection is not as easy as in patients. A typical yield of CSF is 60 μL for an adult rat and 10 μL for a mouse, in some cases with blood contamination. Compared with serum and brain tissues, the CSF has exceedingly low protein content (about 0.8–1 mg/mL in the present study), influencing the choice of proteomics methods. Various methods of proteomic analysis are used to analyze rodent CSF, including isobaric tags for relative and absolute quantitation (iTRAQ), a method widely used owing to its high sensitivity and relatively satisfactory reproducibility. However, an iTRAQ test usually requires at least 50 μL of sample with 5 mg/mL protein concentration, and can only run 8–12 samples in one test. On the contrary, the label-free proteomic analysis uses spectral counting, which is a frequency measurement that uses tandem mass spectrometry (MS/MS) counts of identified peptides as the metric to enable protein quantitation, hence requiring much less sample volume, lower protein concentration, and no additional sample preparation. Using label-free proteomics method, changes in protein expression were analyzed in the CSF of each rat individually in this study.

The timing of CSF collection is crucial. In a LiCl-Pilo chronic epilepsy rat model, the pilocarpine-induced SE works as a severe brain insult, causing recurrent seizures 7 days on average after the onset of SE. The earliest, first unprovoked seizure reported is 5 or 6 days after the SE (29–31). Therefore, the CSF was collected 5 days after the onset of SE in this study to ensure that sample collection was within the epileptogenesis period.

No strictly defined epileptogenesis period exists for patients with encephalitis with concomitant secondary epilepsy. The period between inflammation process and first unprovoked seizure can be regarded as epileptogenesis period. In this study, we collected the CSF right after inflammation of encephalitis had been controlled. In the acute phase, seizures can be displayed as one of the symptoms in some patients with encephalitis. However, whether it should be regarded as secondary epilepsy is difficult to ascertain. International League Against Epilepsy (ILAE) once defined unprovoked seizure in epidemiologic studies as one occurred >7 days after acute CNS infection (32). In this study, only epileptic attacks that occurred during the 2-year follow-up were regarded as unprovoked epilepsy. The similar CSF KNG level of the postacute phase between encephalitis patients with or without an acute phase seizure (Figure 4A) indicated that the elevation of CSF KNG did not depend on the occurrence of a symptomatic seizure.

The CSF contains more than 60% of albumin and several other high-abundance proteins (33, 34). Therefore, some CSF studies reported that depletion of high-abundance proteins helped unmask low-abundance proteins of interest in proteomics (18). In our pilot study, avian polyclonal immunoglobulin Y antibodies (Seppro Rat, sep130, Sigma) were used prior to the proteomic study to remove high-abundance proteins from CSF, including albumin, IgG, fibrinogen, transferrin, IgM, haptoglobin, and alpha-1-antitrypsin. However, it failed to reveal more proteins despite using an extra amount of CSF (data not shown).

In order to screen potential biomarkers for early epileptogenesis, the ideal design would include control rats that experienced SE but failed to develop SRS. However, in our study almost every rat displayed SRS 6 weeks after the onset of SE except only one rat. Besides, the fact that the rat (rat #4) failed to show seizure in 4 days monitoring is not sufficient to decide that the rat had no SRS after SE, because 4 days is too short to establish a “no SRS after SE” group. In this study we video monitored rats' activity continuously and watched the video very carefully and completely. During the chronic period, we monitored each rat for 3–6 days at ~6 weeks after SE. We reviewed over 1,000 h of videos to detect spontaneous seizures. Due to the limitations of the technology and workload, it was not practical to monitor the rats continuously for the 6-week period between the initial induction of SE and the chronic phase of SRS.

To fill this gap, we enrolled encephalitis patients with and without unprovoked seizure during 2 years follow-up. Though the sample size was small, it further confirmed the results from our animal study. We will enroll more patients in future study to re-confirm the results.

### Does a Causal Relationship Exist Between KNG Upregulation in the CSF and Epileptogenesis?

The relationship of kininogen to epileptogenesis has not been explored previously. Although we did not examine whether early stage elevation of kininogen in CSF contributes to the development of spontaneous recurrent seizures, the involvement of bradykinin receptors, including B1 and B2 receptors, are well-established (23, 24, 35–39). Thus, one possible pathway is that bradykinin, which is produced when kallikrein releases it from kininogen, acts on the B1/B2 receptor and contributes or correlates to occurrence of spontaneous recurrent seizures. On the other hand, it is also possible that kininogen may induce epileptogenesis through facilitating BBB damage, because it is found that kininogen-deficient mice shows less severe BBB damage, edema and inflammation formation after thrombosis and ischemic stroke (40). Further studies are needed to examine the effects of KNG on epileptogenesis, including its dynamic change from early stage to chronic period, and the impact of overexpression or downregulation of KNG expressions on development of SRS.

### What's the Source of Elevated CSF KNG?

Another important question was about the source of CSF KNG elevation. The upregulation of KNG levels in the CSF but not in the peripheral blood during epileptogenesis, both in LiCl-Pilo model rats and in patients with encephalitis with concomitant secondary epilepsy, together with the increase in the hippocampal KNG level in LiCl-Pilo rats, suggested that the upregulation of KNG level in the CSF may resulted from increased intra-cerebral synthesis.

A previous study found that during the process of inflammation induced by lipopolysaccharide (LPS), the expression of KNG increased in multiple parts of the brain, including cerebellum, brainstem, hypothalamus, and, most prominently, in choroid plexus (41). Another study showed that inflammatory cytokines TNF-α and IL-1β stimulated KNG mRNA synthesis in mouse choroidal plexus cells (42). The fact that the CSF was mainly secreted by the choroid plexus in the ventricle and the vital role of inflammation and traumatic stress in epileptogenesis (43) might explain the result that KNG experienced a significant elevation in the CSF during epileptogenesis.

We showed in this study that injection of LiCl alone also results in upregulation of KNG when compared to naïve control animals. Lithium has been reported to cause pronounced peripheral pro-inflammatory changes, characterized by an increased percentage of CD3–/CD11+ cells, decreased CD4:CD8 ratio and a sudden increase of IL-1β serum levels (44). These changes were not due to seizure activity and were of similar magnitude as those seen after injection of convulsive dosages of pilocarpine (45). Inflammation has been reported to increase kininogen expression in multiple regions of the brain, most prominently in choroid plexus. On the other hand, LiCl injection causes a rapid permeability increase of the BBB (44). which in turn results KNG leakage from serum to CSF. Thus, it is possible that both inflammation and BBB disruption caused by LiCl injection will lead to KNG increase in CSF. Nevertheless, the elevation of KNG in CSF caused by LiCl injection alone was far less than LiCl-Pilocarpine, reflected by an average ratio of about 1:3 (Figure 2).

There is also concern that KNG may leak from serum to CSF in LiCl-Plicarpine rats, because BBB disruption is a common pathological change both in encephalitis and in epilepsy rat models (43). Thus, further immunohistochemical double straining study is needed to clarify the source of increased CSF kininogen.

Kinins have been reported to be involved in many brain disorders related to secondary epilepsy, including cerebral vascular diseases and neurodegenerative diseases (46), implying that CSF kininogen level may also change in the presence of these disorders. In the present study, we only enrolled encephalitis patients. Secondary epilepsy derives from multiple clinical disorders with probably different underlying epileptogenesis processes. Therefore, how CSF kininogen level is correlated in other CNS disorders, including cerebral vascular diseases and neurodegenerative diseases, remains to be clarified.

## Author Contributions

JZ was involved in study design, data acquisition, data analysis and interpretation, and drafting of the manuscript. XW and LH were responsible for patient enrollment and follow-up. JL and YK helped JZ with collecting rats' CSF and hippocampus samples. SL and QL were involved in study design, data analysis and interpretation, drafting of the manuscript, study supervision, and critical revision of the manuscript for important intellectual content.

### Conflict of Interest Statement

The authors declare that the research was conducted in the absence of any commercial or financial relationships that could be construed as a potential conflict of interest.

## Supplementary Material

Supplementary Figure 1 Proteomic analysis data.

Supplementary Figure 2 Original Western blotting images for KNG level in hippocampus.
