# Supplementary figures and images for: Kininogen Level in the Cerebrospinal Fluid May Be a Potential Biomarker for Predicting Epileptogenesis
Source: Front Neurol. 2019 Jan 31;10:37. doi: 10.3389/fneur.2019.00037 (PMC6371036; doi:10.3389/fneur.2019.00037)

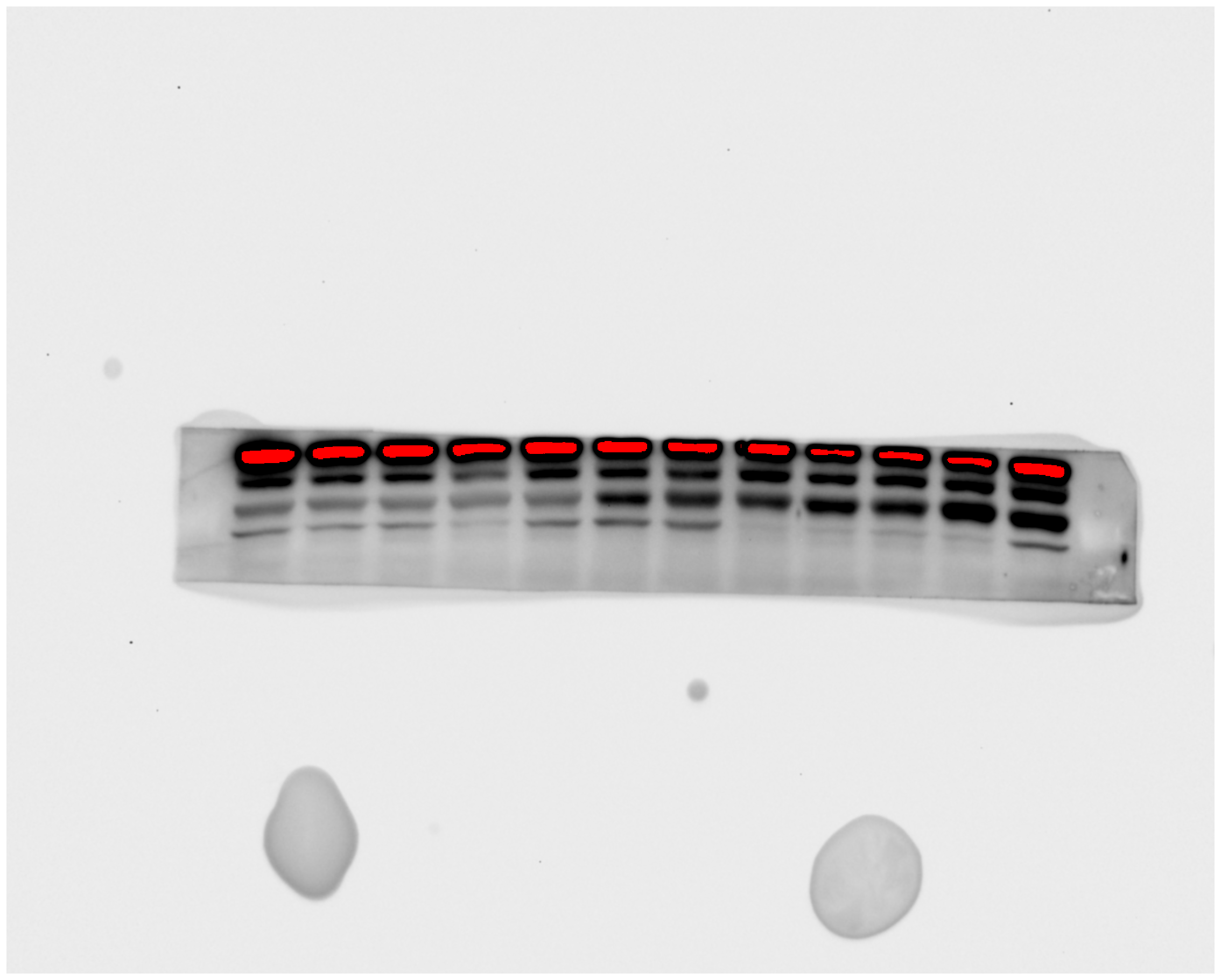

Supplement: Supplementary Figure 1 — Proteomic analysis data. [file Image_1.TIF]

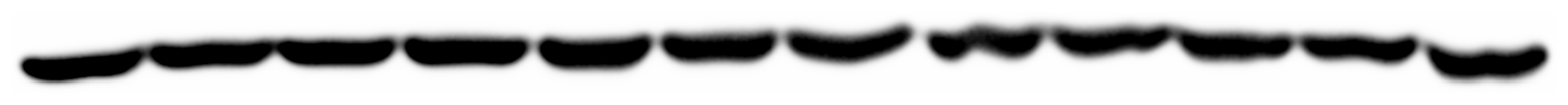

Supplement: Supplementary Figure 2 — Original Western blotting images for KNG level in hippocampus. [file Image_2.TIF]
